# The S2 Subunit of Infectious Bronchitis Virus Affects Abl2-Mediated Syncytium Formation

**DOI:** 10.3390/v15061246

**Published:** 2023-05-25

**Authors:** Shunyi Fan, Yuxi Shen, Shuyun Li, Xuelian Xiang, Nianling Li, Yongxin Li, Jing Xu, Min Cui, Xinfeng Han, Jing Xia, Yong Huang

**Affiliations:** College of Veterinary Medicine, Sichuan Agricultural University, Huimin Road, Wenjiang, Chengdu 611130, China; fanshunyi216@163.com (S.F.);

**Keywords:** IBV, S2 subunit, Abl2, syncytium, cytoskeleton

## Abstract

The S2 subunit serves a crucial role in infectious bronchitis virus (IBV) infection, particularly in facilitating membrane fusion. Using reverse genetic techniques, mutant strains of the S2 locus exhibited substantially different syncytium-forming abilities in chick embryonic kidney cells. To determine the precise formation mechanism of syncytium, we demonstrated the co-ordinated role of Abl2 and its mediated cytoskeletal regulatory pathway within the S2 subunit. Using a combination of fluorescence quantification, RNA silencing, and protein profiling techniques, the functional role of S2 subunits in IBV-infected cells was exhaustively determined. Our findings imply that Abl2 is not the primary cytoskeletal regulator, the viral S2 component is involved in indirect regulation, and the three different viral strains activate various cytoskeletal regulatory pathways through Abl2. CRK, CRKL, ABI1, NCKAP1, and ENAH also play a role in cytoskeleton regulation. Our research provides a point of reference for the development of an intracellular regulatory network for the S2 subunit and a foundation for the rational design of antiviral drug targets against Abl2.

## 1. Introduction

Infectious bronchitis virus (IBV) can impact egg quality through infecting more tissue and spreading to additional organs, including the gastrointestinal tract, kidneys, and oviducts, as well as cause a highly contagious illness [1]. The QX-type IBV virus has a global impact, causing harm to the respiratory, digestive, and genitourinary systems of poultry [2]. The spike protein (including S1 and S2 subunit) is associated with viral antigenicity and plays a role in viral infection and immune activation [3]. The fusion peptides located on the S2 subunit point to its involvement in membrane fusion processes, the creation of viral syncytia, and cytophilicity [4]. The S2 subunit of IBV is essential for the fusion of the viral envelope with the host cell membrane, which permits the virus to discharge its genome into the host cell [5]. IBV mainly enters through clathrin-mediated endocytosis (CME), and viral particles move forward along the classical endosomal/lysosomal pathway, where viral particles can penetrate the cell and cause active cytoskeletal rearrangements [6]. The S2 subunit, which is strongly related to three amino acids near the S2 cleavage site, allows Beaudette strain of IBV to proliferate in Vero cells. [7]. Our earlier studies revealed that S2 mutated strains had dissimilar viral titers, generated irregular syncytia, and showed various levels of pathogenicity in living organism trials [5,8].However, the cause of this phenomenon was not fully elucidated due to a lack of investigation into the role of the S2 subunit. Gaining insight into how S2 subunit mutations affect viral infection can be facilitated through studying the contribution of the S2 subunit to syncytium formation.

The syncytia formed during coronavirus infection enables more efficient virus propagation within and between host cells, thereby enhancing viral replication and spread. Studies of coronavirus infections including IBV, PEDV, and SARS-CoV-2 revealed the possibility of host components, such as CTSB, TMPRSS2, IFITM3, and Abl, being involved in the virus invasion and its passage across membranes [9,10,11,12]. The Abelson tyrosine–protein kinase 2 (Abl2)-mediated actin cytoskeleton regulatory network can play an essential part in the formation of syncytium. Research shows that when Abl2 is inhibited, SARS-CoV and MERS-CoV generate less infectious virus particles, while Abl1 has no effect [13]. Abl kinase inhibitors in IBV restrict cell–cell fusion that produces syncytia via thwarting hemifusion [14]. Abl2 can mediate the complex interaction of the virus–host cytoskeleton during viral particle entry and escape, as well as the ensuing infections. Inhibition of Abl2 during the internalization of human papillomavirus 16 (HPV16) results in the accumulation of aberrant endocytic vesicles [15]. Rift valley fever virus (RVFV) non-structural protein NSs inhibit the transcriptional increase in Abl2 expression and reduce cell proliferation and adherent junction formation [16].

Viruses may take advantage of actin controllers such as Abl to reorganize actin, which can lead to changes in the cytoskeleton that reveal cell surface receptors [17]. The actin-binding domain found at the C-terminus of Abl kinase, which is a non-receptor tyrosine kinase, plays an important role in the control of cytoskeletal motility [18], where the G-actin-binding domain and the microtubule-binding domain are highly conserved across species [19]. Evidence demonstrated that Abl tyrosine kinase activity was boosted through signaling molecules that induced actin rearrangement, indicating that Abl may be controlling upstream and downstream signaling to be involved in managing the actin cytoskeleton [20,21]. Both CRK and CRKL have structural SH2 and SH3 domains, with the SH2 domain mostly being activated via phosphorylation of the upstream Abl family [22,23]. Processes such as pathogen-induced cytoskeletal alterations are mediated via CRK and CRKL [24]. A bridging protein known as abl interactor 1 (ABI1) controls the dynamics of actin [25]. Research suggests that the SH3 structural domain of ABI1 can control Abl-mediated phosphorylation of the WAVE complex, which can be vital for the arrangement of the actin cytoskeleton [26]. NCK-associated protein 1 (NCKAP1) forms the WAVE regulatory complex (WRC) with four other components, including ABI1 [27,28,29]. NCKAP1 interacts with Abl, thereby activating regulatory complexes that influence actin polymerization [30]. ENAH is a cytoskeleton regulatory protein that regulates the actin cytoskeleton and motility. It can aid in cell motility and migration through increasing actin polymerization [31,32].

The function of the S2 subunit and the infection mechanism of coronaviruses remain unknown. This research aimed to uncover the internal pathways through which S2 subunit mutated strains create divergences in syncytial formations. Thus, the initial research goal is to investigate the link between the S2 subunit of IBV and Abl2 in Chick embryonic kidney (CEK) cells.

## 2. Materials and Methods

### 2.1. Plasmids, Cells, and Viruses

**Figure 1:**
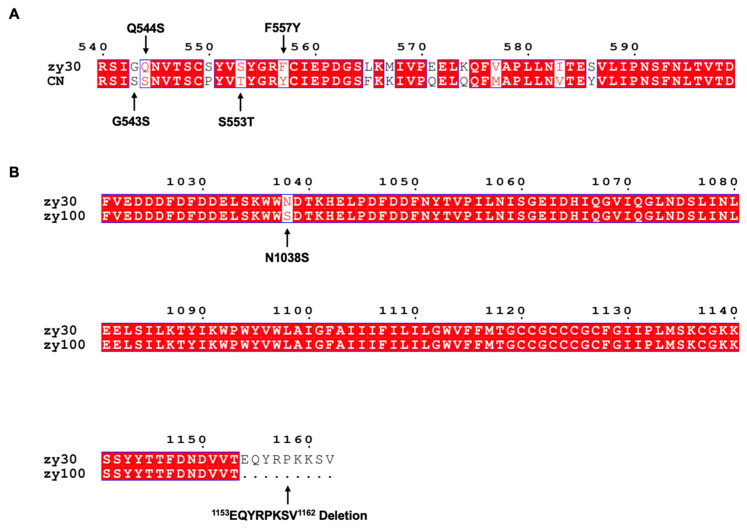
Mutant sites in S2 subunit. (**A**) CN strain of IBV was distinct from zy30, and G543S, Q544S, S553T, and F557Y mutations were implemented to create rSczy3S2_-G543S-Q544S-S553T-F557Y_. (**B**) Differences between zy100 and zy30 were demonstrated, and N1038S mutation and ^1154^EQYRPKKSV^1162^ deletion were applied to create rSczy3S2-N1038S-CT9^∆^. Using ClustalW (https://www.genome.jp/tools-bin/clustalw) (accessed on 16 May 2023) and ESPript 3.0 (https://espript.ibcp.fr/ESPript/ESPript/index.php) (accessed on 16 May 2023), a comparison of amino acid sequences can be made through multiple sequence alignment. Amino acids that are same across all sequences are marked in red, while those that differ from one another are left unhighlighted. This experiment features mutations and deletions that are represented using black arrows.

*E. coli* TOP10 competent cells (Bomed Gene Technology Co., Ltd., Beijing, China) were used to generate eukaryotic expression plasmid, and the pCAGGS-flag was stored in our laboratory. The CEKs were set up as indicated earlier and cultivated in a HyClone Dulbecco’s Modified Eagle Medium (DMEM) supplemented with 10% fetal calf serum (Zhejiang Tianhang BioteChnology Co., Ltd., Hangzhou, China) in a 37 °C incubator with 5% CO_2_ [33]. The Sczy3 strain was discovered in 2009, and was then used to generate zy30 (TCID_50_/mL = 10^4.66^) through 30 consecutive passages of CEKs [34]. A BAC plasmid was used to reconstruct the zy30 genome, producing the rescued strain known as rSczy3 (TCID_50_/mL = 10^4.60^). As previously mentioned, CN is a type of infectious bronchitis virus (IBV) that is unable to infect CEKs. Following a sequence assessment and investigation, CN strain of IBV was used as the benchmark for the mutations G543S, Q544S, S553T, and F557Y in rSczy3, leading to the generation of rSczy3S2_-G543S-Q544S-S553T-F557Y_ (TCID_50_/mL = 10^3.5^) [5]. Successive passages of Sczy3 with CEKs 100 times led to the creation of zy100, which is a variant of zy30 with a distinct S2 subunit that could generate bigger and greater numbers of syncytia when infected with CEKs. Thus, we conducted N1038S substitution and ^1154^EQYRPKKSV^1162^ deletion on rSczy3 to produce rSczy3S2-N1038S-CT9^∆^ (TCID_50_/mL = 10^7.23^) [8]. The graphical representation of the contrast between CN, zy100, and zy30 with regard to amino acid composition, as well as the marked mutation sites, can be seen in Figure 1.

### 2.2. Biological Properties of Recombinant Strains

CEKs were placed at a density of 2.0 × 10^6^ per well in 48-well plates. rSczy3, rSczy3S2_-G543S-Q544S-S553T-F557Y_ and rSczy3S2-N1038S-CT9^∆^ were infected at a multiplicity of infection (MOI) of 2 once the cell density reached 70–90%. Three separate samples of the combination of cell precipitate and supernatant were taken for testing at 12, 24, 36, and 48 h post-infection. After the RNA was extracted, qRT-PCR was employed to measure the replication of the viral genome in line with the reference curve for IBV fluorescence quantification primers IBVF: GGTAAGCATTGAGTGTTGTGGTGA and IBVR: GGTTCTGGTGCCTCTGGAAACA (Sangong Bioengineering Co., Ltd., Shanghai, China) with the formula Y = −3.4168LgX + 10.51 [8], plotting the standard curve of the logarithm of the gene copy number along the *X*-axis and the cycle threshold (Ct value) along the *Y*-axis. After a period of 48 h post-infection, the number of syncytia in each experimental group was documented three times.

### 2.3. Analysis of the Relative Transcript Levels of Host Factors

CEKs were cultivated in 48-well plates under the same environmental conditions. After the cell population had grown to a range of 70–90%, rSczy3 was exposed to MOI = 2, and three samples were taken from the mixture at 12 and 24 h post-infection. The fluorescent quantification primers (Sangong Bioengineering Co., Ltd., Shanghai, China) (Table 1) were created for nine host factors, which were CTSL, CTSB, Abl1, Abl2, IFITM3, ADAM17, TMPRSS2, NRP1, and CD74, that may be associated with S2 function. After RNA extraction, qRT-PCR was used to measure relative fluorescence, and the blank cell set was used as a comparison sample.

### 2.4. Transfection of S2 Protein

CEKs were placed in 48-well plates under the same culture conditions. The S2 subunit from rSczy3 was inserted into the pCAGGS, along with a 4×Flag affinity tag at its C-terminus, meaning that it could be expressed in CEKs using open reading frames (ORFs). The S2 subunit of the construct contains all of the amino acids ranging from the Serine immediately after the S2 cleavage site to the EQYRPKKSV cytoplasmic tail region. The S2 protein expression was assessed through conducting a Western blot with 10–250 kDa Marker (Sangong Bioengineering Co., Ltd., Shanghai, China). Once the cell density reached between 50–60%, transfection was performed using the protocols specified by the TransIntro^®^ EL Transfection Reagent (TransGen Biotech Co., Ltd., Beijing, China). The mock group was transfected with 0.4 μg of pCAGGS-flag per well, while the experimental group was transfected with 0.4 μg of pCAGGS-S2-flag per well. After 24 h of transfection, cells were infected with rSczy3, MOI = 2, and an uninfected control group was also established. At 48 h post-transfection, each cell mixture was taken in triplicate. After the RNA was taken out, the qRT-PCR was employed to figure out the comparative expression of CTSB and Abl2.

### 2.5. RNA Interfering Assay

CEKs were distributed into 48-well plates and, when the cell density of CEKs hit 50–60%, TransIntro^®^ EL Transfection Reagent (TransGen Biotech Co., Ltd., Beijing, China) was used to introduce siRNA-Abl2-1503 (20 pmol per well), while a negative siRNA (20 pmol per well) was given to the control group. The synthetic siRNA sequences (Jima Gene Co., Ltd., Suzhou, China) were as follows: Sense GCCCUCCAAAGGUUUAUGATT, Antisense: UCAUAAACCUUUGGAGGGCTT. Following 24 h of transfection, rSczy3 was inoculated with a MOI of 2 in three replicates. At 24 and 48 h post-infection, cell mixtures were collected for qRT-PCR to detect the relative levels of viral RNA and the relative expression of other factors in the Abl2 pathway, and the effect of Abl2 knockdown on syncytium formation following viral infection was recorded.

### 2.6. Abl2-Mediated Pathways in Mutated Strains

Once the density of CEKs in the 48-well plates reached 70–90%, cells were infected with rSczy3, rSczy3S2_-G543S-Q544S-S553T-F557Y_, and rSczy3S2-N1038S-CT9^△^, using MOI = 2. At 24 and 48 h post-infection, duplicate samples of cell mixes were taken. Table 1 presents the primer sequences (Sangong Bioengineering Co., Ltd., Shanghai, China) for Abl2-related CRK, CRKL, ABI1, NCKAP1, and ENAH. The qRT-PCR was utilized to identify alterations in the mRNA transcript levels of genes that are related to the Abl2-mediated cytoskeletal regulatory pathway.

### 2.7. Affinity Purification and Mass Spectrometry

When the cell density reached 50–60%, pCAGGS-S2-flag (4.0 μg per well) was transfected according to the instructions for the TransIntro^®^ EL Transfection Reagent (TransGen Biotech Co., Ltd., Beijing, China) in four 6-well plates containing CEKs. Following the expression of the S2 viral protein in CEKs for 48 h, lysis was conducted on ice with the addition of NP-40 non-denaturing lysis solution (Biyuntian Biotechnology Co., Ltd., Shanghai, China). Equal quantities of clarified lysates were mixed with flag IgG (TransGen Biotech Co., Ltd., Beijing, China) and the same percentage of mouse negative IgG (ABclonal Technology Co., Ltd., Wuhan, China) as a control. SDS-PAGE gel electrophoresis was conducted at 90 V for 30 min, samples and control gels were recovered, and treated gels were decolored with methylene blue. For LC-MS/MS analysis, the proteins were degummed with 10 mM DTT, 55 mM IAM, and acetonitrile; incubated with 50 mM ammonium bicarbonate for 10 min; and digested with trypsin. Utilized was a Q Exactive HF-X mass spectrometer equipped with a Nanospray FlexTM (NSI) ion source. The ion spray voltage was set to 2.4 kV, the ion transfer tube temperature was set to 275 °C, the mass spectrum was acquired in data-dependent mode, the full scan range of mass spectrum was 350–1500 *m*/*z*, the resolution of the primary mass spectrum was set to 120,000 (200 *m*/*z*), and the AGC was 3106. The parent ion with the highest ion intensity in the top 40 of the full scan was chosen and fragmented using the high-energy collisional cleavage (HCD) method for secondary mass spectrometry. The secondary mass spectrometry resolution was set to 15,000 (200 *m*/*z*), the AGC was 5104, the maximum injection time was 45 ms, and the peptide fragmentation collision energy was set to 27%. The mass spectrometry unprocessed data were produced. The downstream mass spectrometry data were pre-processed, filtered, and classified using the Proteome Discoverer 2.4 software (Thermo Fisher Scientific Inc, Newton Drive, Carlsbad, CA, USA), and the gallus avian source database in Uniprot was chosen for library search. Differential proteins satisfying the criteria could be selected based on the significance thresholds *p* < 0.05, and Fold Change >1.5 or <1/1.5. GO annotation information was obtained from the NCBI database for KEGG pathway analysis and gene function enrichment analysis using differentially expressed genes. jPOSTrepo (Japan ProteOme STandard Repository) contained this portion of the data when using this URL [35]: https://repository.jpostdb.org/preview/1104936592644646cab1a44 (Access key 8553) (accessed on 24 April 2023).

### 2.8. Statistical Analysis

Statistical analysis was conducted using GraphPad Prism 9.3.1 (GraphPad Software, Inc., San Diego, CA, USA). The two-way analysis of variance was used to analyze the data. The statistical significance level was given as follows: * *p* < 0.05, ** *p* < 0.01, *** *p* < 0.001.

## 3. Results

### 3.1. Mutated Strains Have Different Biological Properties

**Figure 2:**
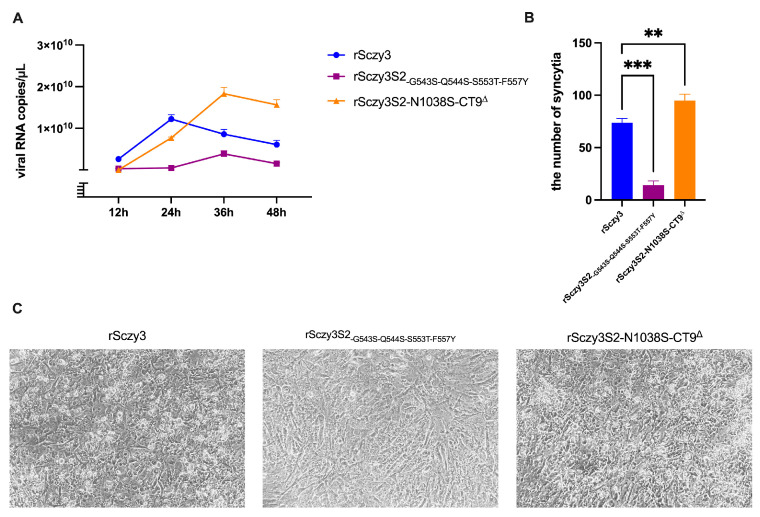
Biological characteristics of mutated strains. (**A**) Viral load of each mutated strain in CEKs was determined at 12, 24, 36, and 48 h post-infection (hpi). (**B**) Number of syncytia produced via recombinant strain is calculated 48 h post-infection (hpi). Results were analyzed using one-way ANOVA, significant distinctions: ** *p* < 0.01, *** *p* < 0.001. (**C**) Photographic depiction of syncytia generated via recombinant strains 48 h post-infection (hpi).

To evaluate the biological characteristics of the mutated strains, we quantified the viral copies and the number of syncytia following infection with rSczy3, rSczy3S2_-G543S-Q544S-S553T-F557Y_, and rSczy3S2-N1038S-CT9^∆^. rSczy3S2-N1038S-CT9^∆^ has the highest viral copies, which is consistent with it causing the most severe cytopathic lesions and forming the greatest number of syncytia. rSczy3 possesses an average ability to proliferate CEKs and can prompt the formation of syncytia and cytopathy. Despite the mutation of rSczy3S2_-G543S-Q544S-S553T-F557Y_ leading to decreased infectivity, it still generates fewer syncytia post-infection (Figure 2). The results suggest that the altered varieties have distinct biological features, resulting in different viral expansion in CEKs and discrepancies in the volume and size of syncytia formed. rSczy3S2-N1038S-CT9^∆^ has the greatest ability to form a syncytium, rSczy3 is in the middle, and rSczy3S2_-G543S-Q544S-S553T-F557Y_ has the least capability.

### 3.2. IBV Infection and Expression of S2 Protein Activate Abl2

**Figure 3:**
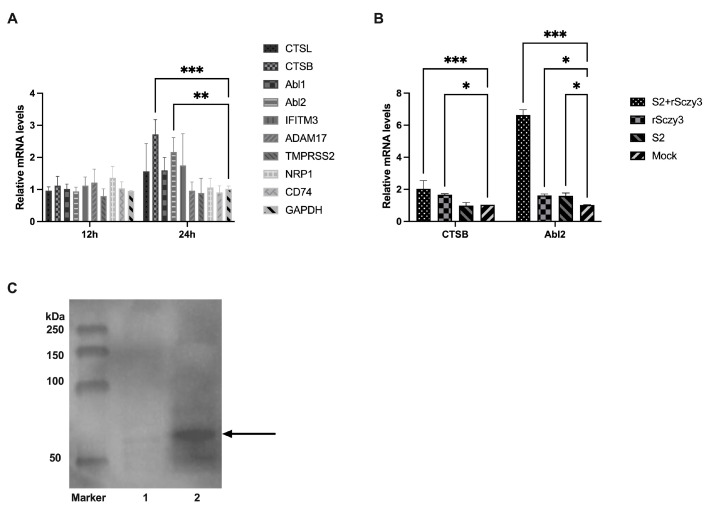
Expression levels of host factors during rSczy3 infection and S2 protein transfection. (**A**) After rSczy3 infection, relative expression levels of nine host variables were assessed at 12 and 24 hpi. (**B**) Under circumstances of rSczy3 infection and S2 protein transfection, relative expression levels of CTSB and Abl2 were assessed, while rSczy3, S2 and Mock groups served as controls. (**C**) During Western blotting results of S2 protein, control group in group 1 was transfected with pCAGGS-flag, and experimental group in group 2 was transfected with pCAGGS-S2-flag. Black arrow points to location of S2 protein. The original graph of the Western blotting can be found in Figure S2. Results were analyzed using two-way ANOVA, significant distinctions: * *p* < 0.05, ** *p* < 0.01, *** *p* < 0.001.

We used the rescued strain rSczy3 to infect CEKs and performed qRT-PCR to evaluate the relative expression levels of CTSL, CTSB, Abl1, Abl2, IFITM3, ADAM17, TMPRSS2, NRP1, and CD74 at 12 and 24 h post-infection. At 12 hpi, no major alterations were observed; however, at 24 hpi, CTSB transcript levels were notably higher in comparison to the control group (*p* < 0.001), and Abl2 transcript levels were significantly elevated (*p* < 0.01). The other host factors, however, did not exhibit major modifications (Figure 3A). Our findings indicate that IBV infection of CEKs for 12 h did not activate transcription of specific host factors, whereas IBV infection of CEKs for 24 h activated transcription of CTSB and Abl2.

To find out whether the expression of the viral S2 protein activates CTSB and Abl2, we used qRT-PCR to analyze the relative expression in the rSczy3 infection, the S2 transfected group, and the mock groups. Western blotting demonstrated the presence of S2 protein, as evidenced by Figure 3C. The findings demonstrated that the expression level of CTSB significantly increased (*p* < 0.001) following the expression of S2 protein and subsequent infection with rSczy3, and that CTSB was significantly raised when infected with rSczy3 alone (*p* < 0.05), while there was no significant change in the group that had only expressed S2 protein. When infected with rSczy3 either on its own or after the expression of S2 protein, the Abl2 gene expression was significantly increased (*p* < 0.05). Furthermore, the Abl2 gene expression was notably enhanced after S2 protein expression and subsequent rSczy3 infection (*p* < 0.001) (Figure 3B). It is evident that CTSB is not induced solely though S2 protein production; however, Abl2 is solely induced in this way. The increase in Abl2 expression as a result of S2 protein expression and IBV infection implies that S2 protein can induce an increase in Abl2.

### 3.3. Abl2 Knockdown Inhibits the Production of Viral Particles and Syncytium

**Figure 4:**
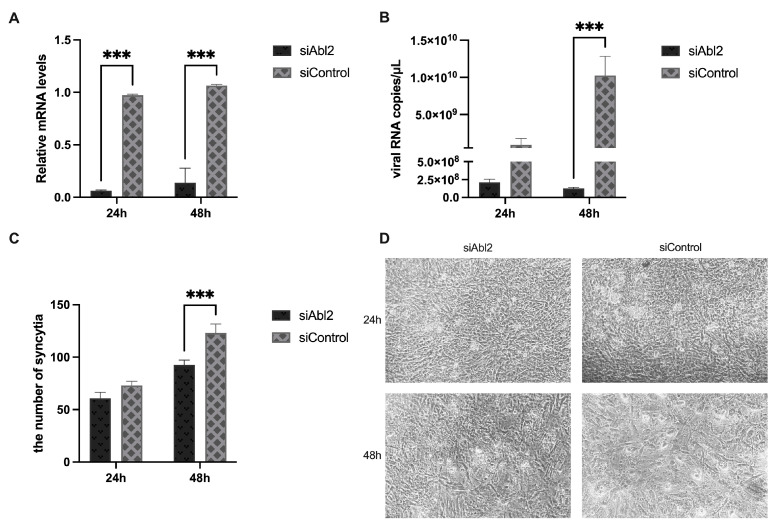
Viral load and syncytium formation following Abl2 silencing. (**A**) Abl2 is inhibited at 24 and 48 h in CEKs. (**B**) IBV viral load at 24 and 48 hpi after Abl2 suppression and subsequent infection with rSczy3. (**C**) Number of syncytia formed via IBV at 24 and 48 hpi following Abl2 suppression and subsequent reinfection with rSczy3. Results were analyzed using two-way ANOVA, significant distinctions: *** *p* < 0.001. (**D**) Formation of IBV syncytia at 24 and 48 hpi.

We conducted Abl2 knockdown experiments to investigate the role Abl2 plays in IBV infection and the formation of syncytia. The results of the RNA interference experiment demonstrated that Abl2 was significantly suppressed (*p* < 0.001) (Figure 4A). The Abl2 knockdown group had a significantly reduced IBV viral load at 48 hpi compared to the control group (*p* < 0.001) (Figure 4B), while there was a significant decrease in the number of syncytia produced in the knockdown group (*p* < 0.001) (Figure 4C,D). Our research suggests that reducing Abl2 expression may lead to a decrease in IBV virus amounts in CEKs and fewer, smaller syncytia.

### 3.4. Multiple Abl2 Signaling Pathways Are Activated in CEKs

**Figure 5:**
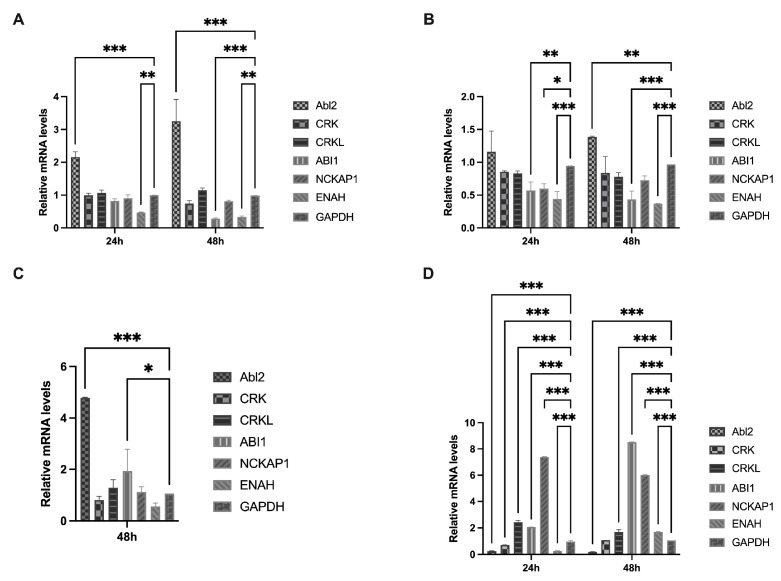
Signaling pathways mediated using Abl2 in CEKs. (**A**) During rSczy3 infection, relative expression levels of signaling pathways connected to Abl2 were measured at 24 and 48 hpi. (**B**) During rSczy3S2_-G543S-Q544S-S553T-F557Y_ infection, relative expression levels of signaling pathways connected to Abl2 were measured at 24 and 48 hpi. (**C**) During rSczy3S2-N1038S-CT9^△^ infection, relative expression levels of signaling pathways connected to Abl2 were measured at 48 hpi. (**D**) During infection with rSczy3 following a RNA interference assay, relative expression levels of signaling pathways connected to Abl2 were assessed at 24 and 48 hpi. Results were analyzed using two-way ANOVA, significant distinctions: * *p* < 0.05, ** *p* < 0.01, *** *p* < 0.001.

We conducted infection experiments using the mutated S2 strain to assess the Abl2-mediated actin backbone regulatory pathway in CEKs, using qRT-PCR to measure the relative expression of host factors. Abl2 expression was consistently increased at 24 and 48 hpi following rSczy3 infection. At 24 hpi, the expression of ENAH was decreased (*p* < 0.01). At 48 h post infection, there was a significant decrease in both ABI1 (*p* < 0.001) and ENAH (*p* < 0.01) expression (Figure 5A). This finding suggests that the rSczy3 infection has a direct impact on actin binding through Abl2, as well as stopping the ENAH regulatory pathway from being activated.

The infection only resulted in a minor increase in Abl2 expression (Figure 5B), as indicated through rSczy3S2_-G543S-Q544S-S553T-F557Y_ (Figure S1). The transcript levels of certain Abl2-related factors were evaluated, and it was found that ABI1, NCKAP1, and ENAH decreased at 24 hpi, with ABI1 and ENAH remaining decreased even at 48 hpi (Figure 5B). This finding shows that the direct regulatory function of Abl2 is necessary for rSczy3S2_-G543S-Q544S-S553T-F557Y_ infection. Furthermore, this mutated strain might not be able to stimulate the ABI1- and NCKAP1-controlled WAVE complex pathway; it may also prevent the initiation of the ENAH control route.

At 48 hpi, rSczy3S2-N1038S-CT9^△^ induced a significant upregulation of Abl2 expression (*p* < 0.001), while determination of the transcript levels of its associated pathway proteins revealed an upregulation of ABI1 expression (*p* < 0.05) (Figure 5C). This finding indicates that the rSczy3S2-N1038S-CT9^△^ infection not only has a direct effect on actin binding through Abl2, but also boosts the expression of ENAH, which is inhibited in the other mutated strain, implying that this mutated strain might have an indirect influence on the control of cytoskeletal motion through activating the ENAH pathway with the help of higher ABI1 levels.

We used RNA interference and qRT-PCR to assess the relative expression levels, thus investigating how host factors affect cytoskeletal rearrangement and syncytium formation following Abl2 knockdown. Our research showed that CRKL expression had a significant rise at 24 and 48 h post-infection (*p* < 0.001). The expression of ABI1 was markedly increased (*p* < 0.001) due to negative regulation, while NCKAP1 expression increased (*p* < 0.001) in conjunction with ABI1, which is involved in the development of the WAVE complex. The expression of ENAH shifted from being reduced at 24 hpi to being increased at 48 hpi (*p* < 0.001) (Figure 5D). It appears that ABI1, NCKAP1, and other WAVE complexes, as well as ENAH, are all indirectly related to the regulation of the cytoskeleton. Furthermore, Abl2 may not be the only factor influencing the regulation of cytoskeletal dynamics through these two pathways.

The Abl2-mediated syncytium formation pathway was not solely determined by Abl2, as the results of the mutated viruses infection assay and RNA interference assay showed that three distinct signaling pathways can alter the cytoskeleton: Abl2, the complex network of ABI1 and NCKAP1, and the ENAH pathway. Furthermore, other host factors may be involved in the regulation of the cytoskeleton following Abl2 depletion.

### 3.5. Bioinformatics Analysis of Mass Spectrometry Results

**Figure 6:**
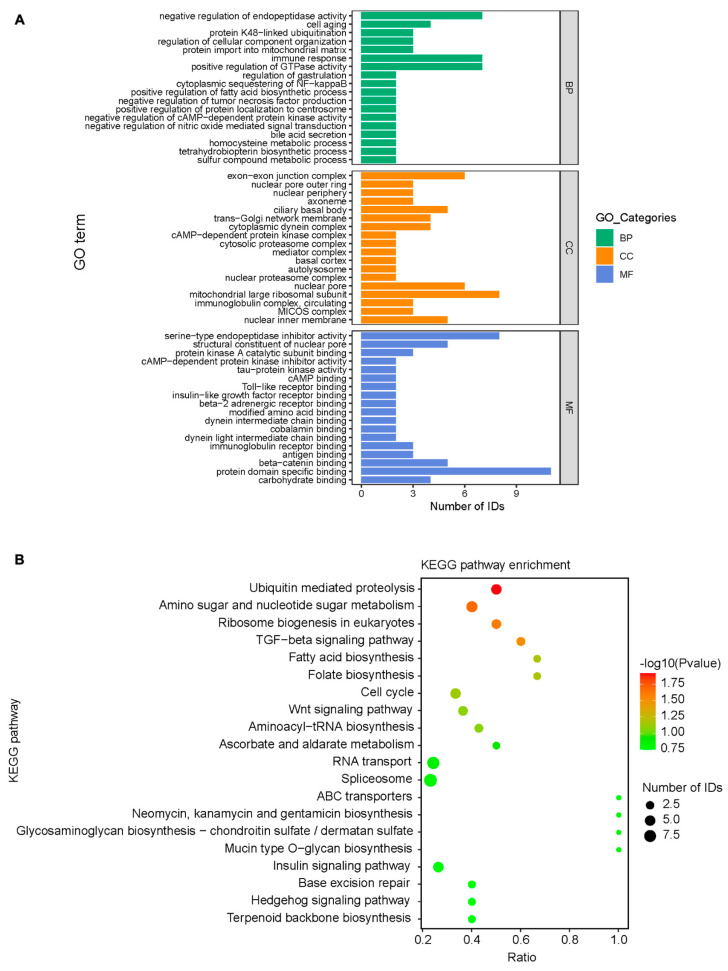
Biofunctional analysis of S2 protein. (**A**) Annotation of differential proteins in mass spectrometry data using Gene Ontology. (**B**) Enrichment of differential proteins in KEGG pathways using mass spectrometry data. Significance criteria: *p* < 0.05 and Fold Change >1.5 or <1/1.5.

The mass spectrometry analysis showed 369 distinct proteins, which were subsequently assigned GO functional annotations and KEGG pathway analysis. S2 protein is primarily involved in intracellular biological processes, such as endopeptidase inhibition, immune response, and GTPase activation; it also conducts protein domain-specific binding, serine-type endopeptidase inhibitor activation, and β-catenin binding. The mitochondrial large ribosomal subunit, nuclear pore, and ciliary basal body are the major cellular components of mitochondria (Figure 6A). S2 proteins could be involved in various intracellular signaling processes, such as the transport of RNA; splicing; metabolizing amino acids, sugars and nucleotide sugars; and the Wnt and TGF-β signaling pathways (Figure 6B).

## 4. Discussion

Infection with infectious bronchitis virus (IBV) in poultry can result in a combination of bacterial infections, causing considerable economic losses in agricultural production [36]. Research into IBV viruses is inadequate, particularly regarding the role of the S2 subunit in the process of infection. Even though IBV is genetically prone to recombination, vaccination is not able to guarantee complete immunity [37]. Thus, understanding how IBV enters and functions in its host organism is essential for gaining insight into its disease-causing properties. We constructed mutated strains of IBV S2 locus using two reverse genetic techniques: rSczy3S2_-G543S-Q544S-S553T-F557Y_ and rSczy3S2-N1038S-CT9^∆^. Our research indicates that Abl2 and S2 do not have a direct connection, while there may be a stimulatory or suppressive relationship between them. Furthermore, S2 protein appears to have a role in the connection between β-catenin and Wnt/β-catenin and TGF-β signaling pathways (Figure 6). The Wnt/β-catenin pathway regulates actin polymerization, where β-catenin facilitates cell-to-cell adhesion through the involvement of microtubules, actin filaments, and intermediate filaments [24,38]. The TGF-β protein were shown to promote the restructuring of cellular skeletons, as well as the dynamic rearrangement of actin filaments [39]. Consequently, the S2 subunit could be responsible for controlling actin and the cytoskeleton, potentially acting as a catalyst for its membrane fusion capability. Abl2 is a potential target for antiviral activity, which can be regulated through competitive inhibitors targeting Abl kinase or engaging ligand molecules via the SH3/SH2 structural domain [40]. Abelson (Abl) kinase inhibitor has antiviral effects in vitro and can prevent viral replication through inhibiting coronavirus fusion [13,14,41].

**Figure 7:**
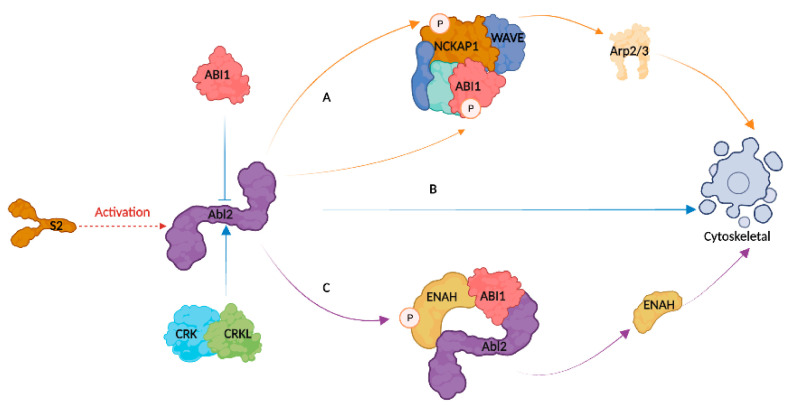
A visual representation of how Abl2 influences regulation. (**A**) Abl2 phosphorylation process regulates a complex containing NCKAP1 and ABI1, which, in turn, affects actin binding activity of Arp2/3 downstream. (**B**) Abl2 has a direct role in binding to actin; however, its activity is controlled through ABI1 and CRK families. (**C**) Combination of ENAH, ABI1, and Abl2, largely driven by ENAH, is able to form a trimer, and connection or separation of these components determines quantity of unbound ENAH. Profilin binds to cytoskeletal regulators, such as ENAH and Arp2/3, in competition with monomeric actin, allowing appropriate monomers to be used to construct actin backbone. It is hypothesized that S2 viral proteins could be involved in either stimulating or suppressing Abl2, therefore playing a role in control of cytoskeleton.

The Abl2 gene encodes a C-terminal region of Abelson kinases, which is responsible for modifying the actin cytoskeleton, controlling cell movement and adhesion, and taking up receptors [42]. Abl2 plays a role in reorganizing the cytoskeleton in response to viral infection, and its purpose is to increase the likelihood of intercellular merging to create syncytia through aiding the early stage of virus–cell membrane merging [14]. The results of transfection of S2 protein and IBV infection revealed that the viral S2 protein caused a rise in Abl2 (Figure 3B), while the suppression of Abl2 had an influence on syncytium formation (Figure 4C,D). It is noteworthy that Abl kinase inhibitors are able to prevent the Beaudette strain of IBV-infected Vero cells from producing syncytia, thus emphasizing the importance of Abl2 [14]. Abl2 predominantly controls cytoskeletal motility through the involvement of five key components: CRK, CRKL, ABI1, NCKAP1, and ENAH (Figure 7). CRK and CRKL, both of which are part of the pathways responsible for cytoskeletal control, are associated with cell adhesion and mobility [23]. Moreover, the SH2 structural domains of these proteins are capable of binding to Abl, a factor which is believed to promote cytoskeletal dynamics [19,22,24]. The WAVE regulatory complex is composed of ABI1 and NCKAP1. ABI1 has the ability to decrease its kinase activity through attaching to the SH3 region of Abl through the PxxP ligand pattern, in contrast to NCKAP1, which can interact with Abl to cause phosphorylation and affect actin polymerization [20,26,30]. The phosphorylation of ENAH, which is caused by the tyrosine residues in Abl, reduces its capability to attach to proteins with SH3 structure, which plays a role in the management of actin. ABI1 acts as a mediator in the formation of the ENAH and Abl2 complex, particularly through stimulating the phosphorylation process [20]. We hypothesized that S2 proteins, while not directly interacting with Abl2, may have an effect on Abl2 and, therefore, be involved in the syncytium formation process, as seen in Figure S1, where different levels of Abl2 activation are observed in mutated viruses.

Alterations of the cytoskeleton can have a considerable effect on viral infection. The SH3, SH2, Tyr Kinase, and FABD actin-binding domains of Abl2 are modulated via ligand stimulation, allowing them to impart mechanical force and take part in clathrin-mediated endocytosis (CME) [43,44]. The entry of IBV into Vero cells is primarily facilitated via CME, which involves the passage of viral particles through the regular endosomal/lysosomal pathway and results in dynamic changes to the cell’s internal structure [6]. Our research suggests that the S2 viral proteins could be linked to Abl2-regulated actin and cytoskeleton activities, instead of interacting directly; this result may shed light on how they manage to execute membrane fusion processes. The WAVE regulatory complex (WRC) relies upon both ABI1 and NCKAP1, and research demonstrated that the Abl family can activate the complex through phosphorylating both of these factors [26,30], thus stimulating the Arp2/3 proteins that bind to actin [45]. The effectiveness of the Arp2/3 complex in regulating actin polymerization is contingent upon the presence of NCKAP1 [27]. The Abl2 protein could be the key factor in the syncytium formation pathway under normal conditions, as evidenced through the rSczy3 and rSczy3S2_-G543S-Q544S-S553T-F557Y_. Despite the mutation to rare amino acids in rSczy3S2_-G543S-Q544S-S553T-F557Y_ at the cleavage site in the front part of the protein, this process may cause a decrease in the performance of the S2 protein and result in a decrease in the formation of syncytia. The mutations G543S, Q544S, S553T, and F557Y, which are all from the non-adapted strain CN of CEKs and are situated near the S1/S2 cleavage site, might be responsible for this activity [5]. The mutations of S2 subunit change its conformation or function, which could impede the activity of ABI1 and NCKAP1 pathway, likely causing the lessening of syncytia (Figure 5B). It is likely that the impairment of the formation of rSczy3S2_-G543S-Q544S-S553T-F557Y_ syncytia causes the CN strain of IBV poor at provoking CEKs lesions. The ABI1 and NCKAP1 pathways were employed to facilitate the assembly of syncytia in Abl2-inhibited experiments (Figure 5D). This evidence suggests that Abl2 may not be the only phosphorylation modulator of the WRC, as the Abl family, Src kinases, and cyclin-dependent kinase 5 can all target phosphorylation sites within it [46].

The Abl family was shown to control actin depolymerization in the cytoskeleton via suppressing ENAH activity and promoting the WAVE complex pathway [47,48]. Our research corroborated previous studies, as our results indicated that rSczy3S2-N1038S-CT9^△^ infection amplified the number of syncytia (Figure 2B,C and Figure 4C). This finding could account for the discrepancy in the production of syncytia between zy100 and zy30. Our research discovered that the N1038S substitution is positioned in the HR2 region, with the ^1154^EQYRPKKSV^1162^ deletion in the cytoplasmic tail being essential for the function of the S2 subunit [8,49]. Alterations in these locations have a direct effect on S2 functioning, which causes Abl2 to become activated and strengthens the binding of actin, as well as causing the ENAH pathway to be indirectly activated. Profilin acts as a binding protein for monomeric actin, and competes with ENAH and Arp2/3 to effectively assign the molecules needed to construct the actin backbone, helping to maintain the stability of the cytoskeletal network [32,50,51]. ENAH knockdown was shown to impair clathrin-mediated endocytosis [52]. Conversely, a higher expression of ENAH was linked to increased cell proliferation, invasion and migration [53], presumably through the modulation of ENAH’s affinity to Profilin, which affects actin polymerization. Studies revealed that the actin-regulating effects of ENAH necessitate the recruitment of WRC [54], while Profilin helps ENAH to assemble actin at Arp2/3, which is downstream of the WAVE complex pathway [55]. It appears that the ENAH pathway is likely to act in a paracrine manner within the cell, likely through binding to Profilin and, thus, increasing the amount of single actin molecules available. Our research indicates that ENAH and ABI1 could potentially lead to a rise in monomeric actin and assist in the regulation of cytoskeletal motility through other pathways (Figure 5C,D).

## 5. Conclusions

Our research indicates that mutated strains influence Abl2-influenced cytoskeleton control. Mutations in the S2 subunit may lead to changes in the growth of viral syncytium. Abl2 works in conjunction with multiple host factors to control the cytoskeleton.

## Figures and Tables

**Table 1:** List of gene primers used for qRT-PCR.

Gene	Accession No.	Forward 5′→3′	Reverse 5′→3′
CTSL	AA495703.1	TGGCTTTGTTGACATCCCTC	AGTGTCCTGCATCAATAGCAA
CTSB	NM_205371.3	CAAGCACTACGGCATCACA	TCCTTCAACTGGGCCGTTC
Abl1	XM_040685978.2	CTGGCAAGAACCTCTACACC	CTTATTGATGGCCTCCCGAA
Abl2	XM_040677820.2	GACAACACGCTCAGTATCACC	CCCTGCCCATTCTTCGAAC
IFITM3	KC876032.1	CGCTGTACGCCAACGTCTGCT	CCGAGGACTTTGCGATCCCT
ADAM17	NM_001008682.2	AGCTCCAAAGATACCTGCAA	CTGCTCTATTTGTATGCCGTA
TMPRSS2	XM_416737.8	ACAGCAATATCTTCCTGCAAC	ACTTCCGTTTTATGTCGCATC
NRP1	NM_204782.1	CCCCACTGATGTTGTTTACAGA	TTCAAATCTCAGCGATACACC
CD74	NM_001001613.2	AAGAACGAGCCATTAATGCTT	CTTCAACCTACACAGGGGTCA
GAPDH	NC_052532.1	GGTGGTGCTAAGCGTGTTA	CCCTCCACAATGCCAA
CRK	NM_001353939.2	CACTCCGCTCCCTAACCTTC	GGCTGTCTTGTCGTAGGCAT
CRKL	XM_415233.8	CATCCACGCAGAACGGACCA	ATCTCCAACCTCTAATGCCAGT
ABI1	NM_001039281.3	GTGCCCATTTCTTCAGGCCAT	TGCCATCAGGTAAGACATACTGC
NCKAP1	XM_040676557.2	GCAGCTTTATTTACCATCCAC	AGTAGTTTTGTCCGTCTCCTG
ENAH	NM_204300.2	CCCTAATGCCTCGACTCCCTC	CTGCCTGCAAGACTGGCTCA

## Data Availability

The data presented in this study are available from the author upon request.

## Supplementary Materials

The following supporting information can be downloaded at: https://www.mdpi.com/article/10.3390/v15061246/s1, Figure S1: Various levels of Abl2 activation occur in mutant strains. Figure S2: The initial display of the Western blotting analysis of the S2 protein.
