# Supplementary material for: The S2 Subunit of Infectious Bronchitis Virus Affects Abl2-Mediated Syncytium Formation
Source: Viruses. 2023 May 25;15(6):1246. doi: 10.3390/v15061246 (PMC10301418; doi:10.3390/v15061246)
Supplement: Supplementary file 1 [file viruses-15-01246-s001.zip › Supplementary file.pdf]

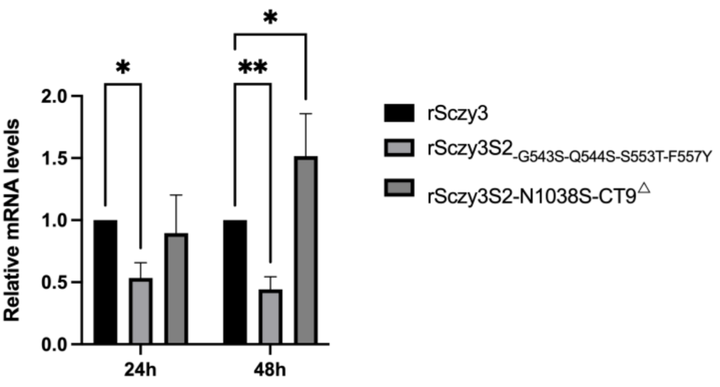

Figure S1. Various levels of Abl2 activation occur in mutant strains. The Abl2 expression levels of the mutant strains were compared with the Abl2 expression of the rSczy3 rescue strain at 24 and 48 hours post-infection with rSczy3, rSczy3S2$_{\text{-G543S-Q544S-S553T-F557Y}}$ and rSczy3S2-N1038S-CT9$^{\Delta}$.

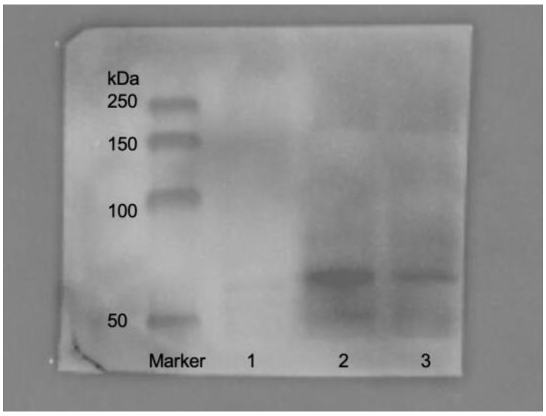

Figure S2. The initial display of the Western blotting analysis of the S2 protein. Group 1 is the control, transfected with pCAGGS-flag, while groups 2 and 3 are the experimental ones, having been transfected with pCAGGS-S2-flag.
